# Effect of intramural myomectomy on endometrial HOXA10 and HOXA11 mRNA expression at the time of implantation window

**Published:** 2013-12

**Authors:** Zohreh Alizadeh, Shamila Faramarzi, Massoud Saidijam, Tahereh Alizamir, Farzaneh Esna-Ashari, Nooshin Shabab, Marzieh Farimani Sanoee

**Affiliations:** 1 *Endometrium and Endometriosis Research Center, Hamadan University of Medical Sciences, Hamadan, Iran.*; 2 *Department of Obstetrics and Gynecology, Hamadan University of Medical Sciences, Hamadan, Iran.*; 3 *Research Center for Molecular Medicine, Hamadan University of Medical Sciences, Hamadan, Iran.*; 4 *Department of Anatomy, School of Medicine, Hamadan University of Medical Sciences, Hamadan, Iran. *; 5 *Department of Epidemiology, Hamadan University of Medical Sciences, Hamadan, Iran.*

**Keywords:** *Myoma*, *Uterine myomectomy*, *HOXA11*, *HOXA10*, *Endometrium*, *Embryo Implantation*

## Abstract

**Background:** HOXA11 and HOXA10 are expressed in endometrium throughout the menstrual cycle and show a dramatic increase during the mid-luteal phase at the time of implantation. The expression of these genes is decreased in women with myomas.

**Objective: **To determine whether myomectomy would reverse HOXA11 and HOXA10 expression, we evaluated the transcript levels of these genes in the endometria of patients before and after myomectomy.

**Materials and Methods:** Expression of HOXA11 and HOXA10 were examined prospectively during the midluteal phase in endometrium obtained from infertile women (n=12) with myoma before and three months after myomectomy. Endometrial HOXA11 and HOXA10 expression were evaluated using quantitative real-time reverse transcriptase-polymerase chain reaction (RT-PCR).

**Results:** Endometrial HOXA11 and HOXA10 mRNAs expression levels (normalized to 18SrRNA) were increased insignificantly in endometrium of patients after myomectomy (p=0.7 and p=0.15 respectively).

**Conclusion:** The results suggest that the alteration in expression pattern of these genes could not account for some aspects of fertility after myomectomy.

This article extracted from M.Sc. thesis. (Shamila Faramarzi)

## Introduction

Lack of preparation of the uterus for embryo adoption, is responsible for nearly two-thirds of implantation failures (1). From the causes of implantation failure, some uterine disease such as reduced endometrial thickness, changes in the expression of molecules involved in implantation and immunology factors that decrease endometrial receptivity have been reported (2). Uterine myoma is one of these diseases. Uterine myoma is the most common, benign gynecologic disorder that is presented in 5-10% of infertile woman (3-4). Depending on its location in the uterus can cause infertility and recurrent miscarriage. Myoma may cause changes in the uterine cavity and endometrium and reduce implantation rate in women undergoing assisted reproductive technologies, compared with women without a myoma (3, 5). 

The outcomes of reproduction become better after myomectomy especially if myoma was the only cause of infertility the differences had more improvements (6). To date there are no molecular data to explain the mechanism behind these clinical observations. It is plausible that myomas adversely affect the endometrium and hence impair endometrial receptivity; however, little is known about the effect of myomectomy on known markers of endometrial receptivity (7). Several genes have been identified that are essential for preparing endometrium to receive embryo, like Homeobox genes, HOXA11 and HOXA10 (8). HOXA10 is a transcription factor that is necessary for embryo implantation. Mice with a targeted mutation of the HOXA10 locus are infertile due to result of failure of implantation (9). 

HOXA10 is expressed in human endometrium during the menstrual cycle, in which its expression is regulated by estrogen and progesterone. Endometrial epithelial and stromal HOXA10 expression levels are up-regulated in the midluteal phase, coincident with the time of implantation (10). HOXA11 homebox genes are the best-known transcription factors participating in implantation. In the mid-secretory phase of a menstrual cycle, which is coincide with the time of implantation, HOXA11mRNA expression is up-regulated in both endometrial glandular and stromal cells in women (11). 

It is shown that expressions of HOXA10 and HOXA11 were decreased in the presence of myoma (7, 12). To determine whether myomectomy would reverse HOXA11 and HOXA10 expression, we evaluated the transcript levels of these genes in the endometrium of patients before and after myomectomy. 

## Materials and methods

This case control study was done from September 2011 to March 2012. The samples were taken at Fatemieh infertility research center of Hamadan University of Medical Sciences. All tissue samples were obtained with full and informed patient written consent. The research protocol was approved by the Medical Ethics committee of Hamadan University of Medical Sciences. 

This study included women of reproductive age, whom had uterine myoma with size greater than 5 cm and were infertile. The subjects were identified prior to surgery, and all subjects underwent myomectomy. At the time of surgery, the following data were obtained: age (under 38), uterine size, obstetric and gynecologic history, medical conditions, medications, surgical history, and last menstrual period. Subjects had not used hormonal medications for at least 3 months prior to surgery. 

(Subjects did not have any other condition previously demonstrated to affect endometrial receptivity such as endometriosis, polycystic ovarian syndrome, or hydrosalpinges). Endometrial tissue biopsies were performed during 19-23 days of a menstrual phase (which is overlapped to mid-luteal phase) before and 3 months after myomectomy using an endometrial suction catheter. Each sample was divided into two portions. The first tissue portion was fixed in 10% formalin for histopathological examination. 

All samples underwent histological evaluation, and normal mid-secretory phase of the endometrium was identified. The second portion was immediately collected in RNA extraction solution (RNX-Plus, Cinagene Company, Iran) and stored at -80ºC until further analysis was performed. Endometrium from subjects before and after myomectomy was evaluated for mRNA expression of HOXA11 and HOXA10.

**RNA extraction**

To obtain total RNA, each sample was placed in 1 mL of RNA extraction solution (RNX- Plus, Cinagene Company, Iran) and homogenized by homogenizer. The cellular lysate was incubated, chloroform 0.2 mL was added, and the samples were centrifuged (17,000 ×g centrifugal force at 4^o^C for 15 min). The clear, aqueous phase was collected and transferred to a new tube, and RNA was precipitated with isopropanol and washed with 75% ethanol. The RNA pellet was air-dried, then resuspended with RNase-free water. From all obtained RNA samples, 2 μl was analyzed using the Epoch Microplatespectro-photometer (BioTek, USA).

**Reverse transcription**

Single-stranded cDNA was synthesized using *Accu Power*® CycleScript RT Pre Mixc DNA Synthesis Kit (Bioneer, Korea) using 1 µg of RNA, according to the manufacturer's protocol. The transcription process included incubation of the reaction mixture at 20^o^C for 30sec, followed by 5 min at 44^o^C, 55^o^C for 30sec and 95^o^C for 5min. The cDNA was stored at -80^o^C until further use for polymerase chain reaction (PCR).

**Quantitative real-time PCR**

PCR analyses were performed using C1000 Thermocycler and CFX96 real time system (BioRad) and QuantiFast SYBER Green PCR Kit (Bioneer, Korea) in a final volume of 25 μl with 10 pmol of each primer. The reaction was incubated at 95^o^C for 5 min, followed by 40 cycles of 15s at 95^o^C, 30s at annealing temperature, 30s at 72^o^C and then fluorescence was measured. Each assay was run in triplicate with each set of primers. Primer pairs for the amplification of cDNA coding for HOXA11 and HOXA10 were designed from the GenBank databases using the AlleleID 6 software and checked for minimum overlap. 

The sequences of primers, accession number and products length are presented in Table I. Annealing temperature were 55.9^o^C and 53.6^o^C and 53.5^o^C for HOXA11, HOXA10 and 18S rRNA, respectively. Specificity of PCR amplifications was verified by a melting curve program (70-95^o^C with a heating rate of 0.5^o^C/s and a continuous fluorescence measurement) and analyzed by electrophoresis on a 1% agarose gel, 1×TBE. 

**Data analysis**

Cycle threshold (Ct) values were obtained through the auto Ct function. Following efficiency correction, the mean threshold cycle (Ct) was calculated and then normalized to the reference gene (18S rRNA) using delta (Δ) Ct. Changes in relative expression were calculated using the 2^-∆∆ct^ method (13). The specific transcripts were presented as n-fold change relative to pre-myomectomy level. 

**Ethical considerations**

All tissue samples were obtained with full and informed patient consent. The research protocol was approved by the Medical Ethics committee of Hamadan University of Medical Sciences.

**Statistical analysis**

ΔCt was reported as means±SEM of three independent experiments. Values of p<0.05 were considered significant. Results were analyzed using student’s *t* test for comparison between pre and post operation. 

**Table I:** Primer sequences used in PCR

**Gene**	**Primer sequence**	**Product size (bp)**	**Accession no.**
HOXA10	Sense: 5’ CTCCCACACTCGCCATCTC 3’	187	NM_021192.2
	Anti-sense: 5’ CAAACCCAGCCCAGTCAGG 3’		
HOXA11	Sense: 5’ AATGGCTGTGGAGTGTGG 3’	226	NM_021192.2
	Anti-sense:5’ CTCTCAGGCTCTTGGAAGG 3’		
18S rRNA	Forward: 5’ GTAACCCGTTGAACCCCATT 3’	152	X03205
	Reverse: 5’ CCATCCAATCGGTAGTAGCG 3’		

## Results

**Figure 1:**
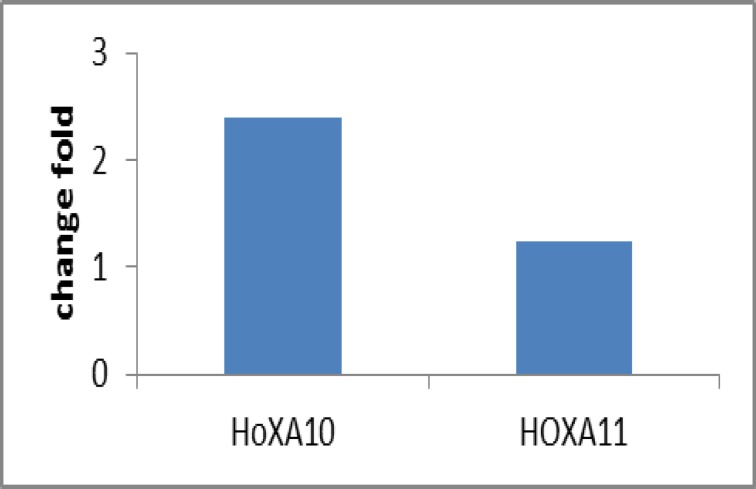
Change fold of HOXA10 and HOXA11 mRNAs after myometomy. The fold-chang of HOXA10 and HOXA11 after myomectomy wasn’t signiﬁcantly different

The clinical characteristics of infertile patients (n=12) with myoma are summarized in Table II. In this study, the mean±SD age of all subjects with myoma was 31.7±2.65 years. The mean size of subjects was 4.02±1.64×6.04±1.44 centimeters. An increase in endometrial HOXA11 expression was seen in 8 of the 12 postmyomectomy endometrial samples compared with corresponding premyomectomy samples (ΔCt values: 11.79±3.06 versus 12.10±1.98; p=0.7). HOXA11 levels were increased by 1.24 fold after myomectomy (Figure 1). 

Endometrial HOXA10mRNA expression (normalized to 18S rRNA expression) was higher in uterus with postoperative than preoperative samples but this difference was not also significant. (ΔCt values: 27.50±2.51 and 28.77±3.60, respectively; p=0.15). An increase in endometrial HOXA10 expression was seen in 9 of the 12 postmyomectomy endometrial samples. HOXA10 levels were increased by 2.39-fold after myomectomy (Figure 1).

**Table II:** Demographic characteristics of infertile patients with myoma

**Patient number**	**Age**	**Size of myoma (cm)**	**Type of myoma**
1	37	6×6	intramural
2	36	4.6×6	intramural
3	35	2.3×4	intramural
4	36	4.8×7	intramural
5	33	6.5×5.3	intramural
6	37	2.5×6	intramural
7	30	3.8×9	intramural
8	31	5.6×42	intramural
9	35	2.6×7	intramural
10	31	1.5×7	intramural
11	37	4×5	intramural
12	37	5.5×2	intramural

## Discussion

Endometrial changes resulting in its receptivity requires a delicate coordination of a number of different events at structural, cellular and molecular level. "Impaired endometrial growth and differentiation may be an important factor contributing to infertility and recurrent pregnancy loss. It has been reported that 50-75% of the pregnancy loss are due to failure in implantation"(3). Several hypotheses have been proposed to explain the association between fibroids and infertility, including potential effects on sperm transport, uterine contractility, endometrial changes, and implantation (14-15). 

Also multiple studies, including a recent meta-analysis, have shown the presence of non-cavity-distorting intramural fibroids is associated with adverse pregnancy outcomes in women undergoing IVF treatment (16). Therefore the overall beneﬁt of myomectomy before IVF in improving reproductive outcome; has been recommended as a therapeutic option for patients with myoma (17). Several genes have been identified as molecular markers of endometrial receptivity: like HOXA10 and HOXA11 (8). 

"Homeobox (Hox/HOX) genes encode transcription factors that mediate embryonic development. In the human, HOXA11 is expressed in endometrial glands and stroma throughout the menstrual cycle" (18). HOXA11 is essential for implantation in the mouse as shown that disruption of this gene results in sterility. The ovulation takes place in these animal but their embryos fail to implant (12, 19). HOXA10 is expressed in both endometrial epithelial and stromal cells, where it likely subserves the different physiological functions of the sex steroids (20). In the mid luteal and late luteal phases, epithelial HOXA10 expression is sustained at high levels despite the decline in Pregesteron-receptor concentrations. 

Endometrial epithelial HOXA10 expression is driven by both stromal paracrine factors and also HOXA10 auto-regulation; HOXA10 also regulates its own expression in endometrial epithelium (21-22). The expression of HOXA10 and HOXA11 genes were decreased during the secretory phase of endometrium in some nonoptimal conditions such as in adenomyosis, endometriosis, myoma idiopatic infertility (23). To determine whether myomectomy would reverse HOXA11 and HOXA10 expression, we evaluated the transcript levels of these genes in the endometrium of patients with intramural fibroid before and after myomectomy. According to our results, HOXA10 and HOXA11 mRNAs expression in endometrium of post-myomectomy shows a higher level compared to pre-operative; however, the differences failed to reach statistical significance. 

Rackow and Taylor investigated the effect of uterine leiomyomas on these markers of endometrial receptivity HOXA10 and HOXA11. The expressions of HOXA10 and HOXA11 were significantly decreased in the presence of submucosal myoma. They found no significant changes in expression of these genes in presence of lyomyoma (7). Our study performed on patients with intramural (liomyoma) and the results are agreed with Rackow and Taylor study. Rackow and Taylor also suggested that submucosal leiomyomas cause global changes in endometrial instead of local limited to the site of myoma (7). In our study endometrial tissue biopsies were performed randomly through part of endometrium. Although intramural myoma (with no change of endometrial cavity) were not associated with a significant change in HOXA10 and HOXA11 gene expression, a decreased endometrial HOXA10 mRNA and stromal protein expression was noted in this group as compared to control group (3, 7, 24). 

The global effect of submucosal myomas on endometrial receptivity was proposed to be mediated by a diffusible signaling molecule that originates from the myoma. The authors suggested that the same signaling pathway might also exist from intramural leiomyomas to the endometrium. However, because of the greater distance and hence low concentration, this signaling molecule causes a less marked effect on markers of endometrial receptivity compared to that seen with submucosalmyomas (4).

In the current study myomectomy was performed for infertile women who had myoma (with average size 4.02±1.64× 6.04±1.44) with distorting the endometrial cavity and who had repeated in vitro fertilization-embryo transfer failure over three or more cycles. Horcajadas *et al* using functional genomics of the endometrium during the window of implantation suggested that endometrial receptivity should not be affected by the presence of intramural leiomyomas not distorting the uterine cavity (24). In agreement with this study, both HOXA10 and HOXA11 genes expression were insignificantly increased in the endometrium after removal of myoma. 

Although the increased expression level of these genes were not significant after 3 months. In our study the post-myomectomy endometrial biopsies were performed three months after operation in mid-menstrual cycle. This strategy was employed as the normalization of the endometrium has been accepted as happening after three cycles of continued treatment in certain disorders, such as dysfunctional uterine bleeding (25-26). Performing sequential monthly biopsies to determine the progressive change would have been ideal, but this was not possible due to ethical concerns.

## Conclusion

This study provides evidence that removal of intramural leiomyomas not affecting the expression pattern of HOXA10 and HOXA11 endometrial genes.
